# Making Vector-Borne Disease Surveillance Work: New Opportunities From the SDG Perspectives

**DOI:** 10.3389/fvets.2019.00232

**Published:** 2019-07-16

**Authors:** Marieta Braks, Giorgia Giglio, Laura Tomassone, Hein Sprong, Teresa Leslie

**Affiliations:** ^1^Center for Zoonoses and Environmental Microbiology, National Institute for Public Health and the Environment, Bilthoven, Netherlands; ^2^Dipartimento di Scienze Veterinarie, Università degli Studi di Torino, Torino, Italy; ^3^Eastern Caribbean Public Health Foundation, Oranjestad, Bonaire, Sint Eustatius and Saba

**Keywords:** vector-borne disease, surveillance, one health, sustainable development goals, dengue, Lyme borreliosis

## Abstract

Surveillance of vector-borne diseases (VBDs) exemplifies a One Health approach, which entails coordinated, collaborative, multidisciplinary, and cross-sectoral approaches to address potential or existing health risks originating at the animal-human-ecosystem interface. However, at the intervention stage of the surveillance system, it is sometimes difficult or even impossible to act. The human dimension of VBD control makes them wicked problems requiring an interdisciplinary systems approach beyond the One Health domain. Here, we make a case that the agenda of the UN Sustainable Development Goals (SDGs) can offer new opportunities to address these issues. The health of the population is a concern to us all and is more or less related to all 17 SDGs. The SDGs can provide a common language by which the interests of various stakeholders can be matched and the challenges that society faces identified, studied, and alleviated. To illustrate, the control and prevention of two VBDs, dengue and Lyme borreliosis, were selected and related to specific SDGs. Further, we use the framework proposed by the International Council of Science to: (1) show synergies and trade-offs between the various SDGs; and (2) present SDG 3 to identify policy that can be related to prevention. Engaging in an integrated approach will confront stakeholders with various viewpoints and through these oppositions, innovation can be nurtured. By adhering to the SDG agenda, we present policy advice including new opportunities for vector-borne disease control to reach its own health goals, while simultaneously supporting other sustainable development goals.

## Vector-Borne Diseases and One Health

Vector-borne diseases (VBDs) are a broad and varied group of diseases with the common denominator, that the pathogen must be transmitted through an arthropod vector. Human VBDs are often zoonoses. The interaction between vertebrate host, vector and pathogen translates into an intricate transmission dynamic; changes of these factors can lead to VBD's introduction in a new area, expansion in an infected area, or re-introduction in a past-infected area (1). The intricate cycle of VBDs and the impact of external drivers (e.g., global warming, immigration, urbanization, globalization, and inequity) make surveillance the linchpin of an integrated fight against them.

As a general rule, surveillance systems provide information for action, through a feedback structure that includes monitoring and intervention activities. Surveillance feedback systems are common in the health sector. In Europe, for example, countries receive signals that the measles incidence has been increasing, while the vaccination coverage has been decreasing. This triggers a call from the European Centre of Disease Control for an increased effort to encourage citizens to get themselves and their children vaccinated in order to decrease the disease burden (2, 3). Although multi-facetted, the mentioned surveillance for measles and other surveillance health systems can suffice to be monosectorial, while others benefit from involving stakeholders from other sectors. Most VBD surveillance systems are clear examples of the latter, even for the three vaccine-preventable arboviral zoonoses, yellow fever, Japanese encephalitis, and tick-borne encephalitis. While outbreaks of these diseases can be prevented and controlled by massive vaccine campaigns, the infection pressure from the animal reservoir host is unaffected by these vaccines. Thus, integrated surveillance systems are required to closely monitor the situation.

We previously developed a general framework for VBD surveillance feedback systems, including research, monitoring, decision-making, and interventions (4, 5). Here, the ultimate design of a surveillance system is closely related to the context in which the disease occurs: presence/absence of vector, pathogen and human cases define different risk scenarios regarding disease's introduction, establishment, and spread (5). Thus, different contexts call for distinct interventions. For example, in the Netherlands where Lyme borreliosis is endemic, interruption of transmission is necessary to control disease burden. In cases, where, for example, the vector is not present, but pathogens are frequently introduced by viraemic travelers, prevention of the establishment of invasive vector species takes priority. In cases in which there is no vector, pathogen, and human cases, it is advised to prepare and be vigilant by closely following the early warning signs from neighboring areas^1^

VBD surveillance, depending on context, is generally composed of monitoring the disease, pathogen, vector, and environment (including climate) allowing authorities to make informed decisions on whether or not to intervene (4). As previously mentioned, surveillance entails gathering information for action and must not be mutually exclusive of the actual intervention activities; they are dependent, interrelated, and part of the circular system of surveillance, in which everything is inter-connected. Depending on the VBD, surveillance is traditionally phrased as a concern of human health (HH), veterinary health (VH), and environmental health (EH), individually or in combination. For example, in the case of bluetongue, VH is primarily responsible, while in canine leishmaniosis HH and VH will be primarily involved. As for West Nile fever, HH, VH, and EH need to all work together.

The recognition that the health of people is intimately connected to the health of animals and the environment is the foundation of the One Health concept. It was officially adopted by international organizations and scholarly bodies in 1984, but has only become commonplace as an umbrella term, capturing an integrative approach to human, animal, plant and environmental health, since 2000 (6). Multi-sector collaborations ensure better preparedness and contingency planning, more efficient and effective surveillance systems, cost-sharing between sectors according to the benefits of control, increased health equity and improved sharing of logistics and costs for service provision (5). One Health initiatives have been well developed and implemented for monitoring and providing early warning of vector-borne zoonoses such as West Nile fever (7, 8), Rift Valley fever (9), leishmaniosis (10), and Chagas (11). However, interdisciplinary health approaches often fall short in the prevention and control of VBDs, because they involve challenges beyond the health domain.

## Wicked Problems and Integrated Solutions

The prevention and control of VBDs can be described as a wicked problem. A wicked problem is a societal problem that is so complicated it requires social, ecological, and economic tradeoffs in order to address the situation. Moreover, because of strong interdependencies, the effort to solve one aspect of a wicked problem may reveal or create other problems. Wicked problems are difficult to define and delineate from other and bigger problems and when they are not solved once and for all, tend to resurface. Unlike the so-called tame problem, wicked problems cannot be solved by one field alone. There is often no technical solution, it is not clear when they are solved, and they have no right or wrong solution that can be determined scientifically. In fact, a scientific approach, which gathers data, analyzes data, proposes and implements solutions, has a high rate of failure. For wicked problems, governance must rely on the collective judgement of various stakeholders involved in an integrated process that is experimental, interactive and deliberative (12, 13).

Integrated approaches are not new to combating VBDs. Ever since 1897 when Sir Ross proved that malaria was transmitted by *Anopheles* mosquitoes, medical approaches were supplemented with vector control to combat the burden of VBDs. Vector control entails the physical elimination of vector breeding sites and reduction of contact between host-vector, as well as the chemical and biological control of the vector population. Shortly after World War II, when synthetic insecticides for agricultural use became widely available, control strategies combining both chemical and biological agents against insects were developed. The publication of the book entitled Silent Spring by Rachel Carson in 1962 called for Integrated Pest Management (IPM) for the use of biocide to balance agricultural yield and environmental health. The World Health Organization (WHO) promotes, initially for malaria control, the application of Integrated Vector Management (IVM) strategies to rationalize decisions for the optimal use of resources for vector control (14).

There are many obvious advantages of integrated approaches, but often the word integrated is used, but not practiced. For example, with VBDs, there is a prime focus on vector and the pathogen, while people continue to be defined as passive actors. The fact is they are not. People do vector control, create environments that are conducive for the vector and behave in ways that decrease (e.g., use of protective clothing against tick bites) or increase (e.g., frequenting/inhabiting tick-infested areas) their contact rate with vectors. The range of what people do can be measured on the local level by investigating the roles people play in making decisions to cut vector programs leading to a lack of human resources and materials. In the case of mosquito-borne diseases, people may allow water to settle around their premises allowing females to lay their eggs and ultimately increase mosquito density. The impact of human decisions plays a role in increasing temperatures, which allow vectors to invade new territories and thrive in regions spreading the pathogen.

Thus, central to the control of VBDs are people and their environment. Climate change, rapid unplanned urbanization, poor water and waste management are all variables that account for the rapid dispersion of the vectors carrying pathogens. Land use changes such as urban green and blue climate adaptations to alleviate urban heat islands or restore the connectivity of natural areas for biodiversity purposes facilitate the expansion and exchange of vectors and the pathogens they carry (15). Globalization has allowed the international dispersion of *Aedes albopictus* and has been responsible of two chikungunya outbreaks in Italy (16, 17). Many of the challenges underlying these variables involve the decisions that are made on the local, regional, national, and global level. Furthermore, people make everyday decisions about the control of vectors. A study in Sint Eustatius, Caribbean Netherlands, revealed that people did not distinguish between mosquitoes generally, and the *Aedes aegypti* mosquito in particular. Furthermore, the mosquito was also defined as more of a general pest as opposed to a disease threat and mosquito-borne diseases were not considered a primary health concern (18). Given this scenario, VBDs are not a pest problem, they are a people problem. This does not mean that people are the problem, but that the beliefs of people are important to finding a solution. Thus, to find a solution a dialogue between expert and lay perceptions is fundamental to identify “what” the problem is. If it is not the VBD, perhaps it is the morbidity or the financial costs associated to disease. With wicked problems there are no trivial solutions. Perhaps VBDs require a negotiation of perceptions to reframe the problem. Often there is a clear equity aspect that may be more concerning than the disease itself.

The level and nature of integrated approaches depends largely on the context of the issue of concern. Smart choices need to be made to connect with the appropriate stakeholders. Thus, important challenges of integrated approaches are on the one hand not to forget particular stakeholders, and on the other hand, to make sure that your theme is not forgotten by other sectors. For example, the social sciences as a field, is capable of investigating human behaviors but are often not included throughout the process of VBD control and prevention. According to Reidpath et al. (19) VBDs “represent a rich and dynamic interplay between vector, host, and pathogen which occurs within social, physical, and biological contexts. The overwhelming sense, however, is that research into neglected tropical diseases [NTD] comprising seven VBDs (20) is a biomedical endeavor largely excluding the social sciences (19).” They continue “The evidence from the literature, however, is that there is little investigator driven social science to speak of in the NTDs, and a similarly poor presence of interdisciplinary science. Without this, our understanding and management of NTDs is inevitably reduced to a strategy that relies on a repetitive, reductionist, flat-world science to overcome an acknowledged complex system.”

If VBDs represent a wicked problem “the approach often referred to as the scientific method is not the best way to approach them” it is no wonder that, to a large extent, the control and prevention of VBDs has failed. Approaches to management and governance of VBD control and prevention often fail to appreciate such cross-sectoral feedbacks. A change in perspective is necessary. The system approaches principle that has many origins (21) places individual system elements in their environments and observes the relationships between them. Adoption of systems approaches allows for the anticipation of unexpected negative or positive consequences and formulation of potentially wiser interventions (13, 22).

## Sustainable Development Goals

Truly integrative approaches benefit from active involvement of various stakeholders across sectors. Interactions between stakeholders should be bidirectional and some may result in opposing or contradictory views. The Sustainable Development Goals (SDGs) provide a framework for such an integrative approach. The United Nations stated: **“**The Sustainable Development Goals are the blueprint to achieve a better and more sustainable future for all. They address the global challenges we face, including those related to poverty, inequality, climate, environmental degradation, prosperity, and peace and justice. The goals interdepend and interconnect with each other in order to leave no one behind^2^” What the sustainable development goals illustrate is that a healthy population is not just a concern for the public health department and/or the health care system. The health of the population is the concern of all and is more or less related to all 17 SDGs. However, SDGs go further than advocating “health in all policies” (23) or One Health approach (24). Health is not the ultimate all-embracing single goal concerning the world; no SDG has hierarchy over the others. What further must be remembered about the SDGs is that no individual goal is the sole responsibility of a single sector or discipline. It calls for a multi-sectorial and transdisciplinary approach where the traditional boundaries separating sectors and disciplines bend and overlap. Communities also need to be empowered so that they become actors in constructing futures that are equity based. The SDGs should provide a common language so that the interests can be matched and the challenges facing society identified, studied and alleviated. The involvement of these multiple parties will allow top down approaches to align with those from the bottom up so that addressing challenges outside one expertise will no longer be perceived as imposing your problem on third parties.

Especially in the tropical southern hemisphere, many of the drivers and manifestations of NTD's and malaria are poverty (SDG1) and social inequality (SDG 10) (25, 26). As mentioned previously, social factors associated with infectious disease outbreaks are often neglected and the aftermath is ignored. These factors can affect outbreak severity, its rate and extent of spread, influencing the welfare of victims, their families, and their communities (27). In addition to this is the inclusion of the private sector that can provide decent work and economic growth (SDG 8) and innovation and infrastructure (SDG 9). It could become their role to think about ways to alleviate inequalities by assisting in the development of social entrepreneurship that could foster innovation and actively manage and control VBDs. Also not to be forgotten is the community's role in good surveillance. With the community, it may be possible to enter from a different direction as opposed to the entrance through the VBD itself. The question could be how do we reduce inequalities in society (SDG 10) and promote gender equity (SDG 5)? Is it a possibility that if the community is empowered they may be more willing to play an active role in various activities that can improve sustainability? What processes can be used that will work to galvanize necessary moral commitment on the part-of people, institutions and international organizations to address issues of poverty and sustainable livelihoods? How do VBDs fit within this larger issue of empowerment and how can empowerment strategies be used to involve communities in surveillance efforts? How can focusing on community empowerment assist in developing institutional solutions, sustainable approaches and partnerships (SDG 17), that will lead to effective surveillance practices and ultimately protect the health and well-being of the greater populations (SDG 3)? It is empowerment, which provides communities with the tools to engage in the interactions between ecosystems, political and socio-political change that affect their lives adversely (28). VBDs are one example to this and such thought patterns are required to develop a truly integrative approach.

The Millennium Development Goals for 2000–2015 that preceded the SDGs *de facto* focused on low and middle income countries, because they had to take the furthest leap. The SDGs, however, explicitly address all countries to participate. No country has achieved all goals yet. The situation in Europe and the United States differs from that in tropical countries in the southern hemisphere where resources are often lacking. Also in countries, where human resources, proper infrastructure and capacity to provide proper surveillance are scarce, control of VBDs often falls short. The recent outbreak of murine (endemic) typhus or flea-borne typhus in Los Angeles county, California (October, 2018) illustrates how VBD's are a wicked problem. Murine typhus is a disease caused by a bacterium called *Rickettsia typhi* and is spread to people through contact with infected fleas. People get sick with murine typhus when infected flea feces are rubbed into cuts or scrapes in the skin. While in most areas of the world, rats are the main animal host for fleas infected with murine typhus, feral cats may also serve as a host as well. In October 2018, forty individuals in Los Angeles County had become ill due to this infection. Interestingly, all cases had a history of living or working in the downtown Los Angeles area where inhuman conditions are increasing due to the county's expanding homeless population. This scenario fits very well into the One Health model as there is a link between animal, human and environmental health. However, while treatment for the disease is available (SDG 3), the underlying environmental conditions influencing this outbreak are rooted in social and economic conditions and those individuals who exist at the fringes of society are the most susceptible (SDG 1, SDG 6, SDG 8, SDG 10). Because of the infectious nature of typhoid, the poor and disenfranchised in urban areas (SDG 11) will not remain the only ones impacted as infectious agents do not know socio-economic borders. This becomes an issue that is beyond the traditional realm of public health and individuals from multiple arenas should be involved in resolving this wicked problem in the long run. If this does not happen, the epidemic may be controlled, but only to arise in the not too distant future.

## Implementation of SDG's

The United Nations' 2030 Agenda for Sustainable Development, underpinned with 17 SDGs and 169 targets, was adopted in September 2015. Policymakers face the challenge of implementing the SDGs simultaneously with the aim of achieving progress across the economic, social and environmental dimensions worldwide (22). Social sciences are essential for surveillance, not only because they can understand the social context of diseases, but also because they help us to understand the social context of surveillance plans. As mentioned earlier, there are many obvious advantages of integrated approaches, but not often practiced successfully, because it is just plain difficult. The International Council of Science recognized this challenge and provided the following framework to get from science to implementation of SDGs (22):

“The framework identifies categories of causal and functional relations underlying progress or achievement of goals and targets. The scale ranges from −3 to +3, from instances where progress on one target acts to cancel progress on another to where progress on one goal is inextricably linked to progress on another. Complementing the scale is a number of key dimensions (time, geography, governance, technology, directionality) that describe the interactions and define the context in which they occur. Most interaction scores depend on these dimensions and putting in place the right policies and technologies might shift the score to a more positive one. To be more specific, positive interactions are assigned scores of either +1 (“enabling”), +2 (“reinforcing”), or +3 (“indivisible”), while interactions characterized by trade-offs are scored with −1 (“constraining”), −2 (“counteracting”), and −3 (“canceling”). Thus, the magnitude of the score, in whichever direction, provides an indication of how influential a given SDG or target is on another.”

**Figure 1:**
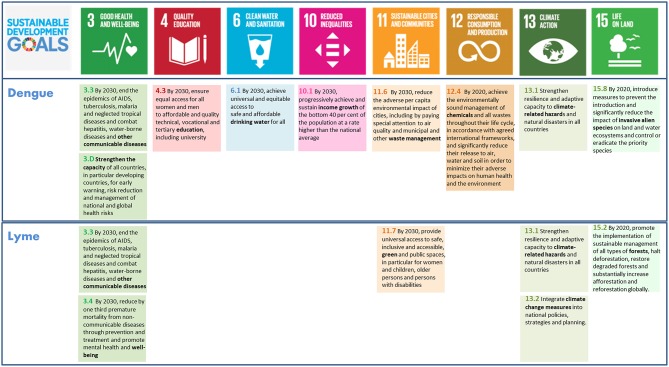
Overview of the key sustainable development goals (SDG 3, 4, 6, 10, 11, 12, 13, 15) and targets related to prevention of dengue and Lyme borreliosis. Note that SDG 1, 2, 5, 7, 8, 9, 16, and 17 are missing. For overview all SDGs and targets see (22).

Below, we present a discussion of how to apply this described framework to integrated vector management for dengue control and the prevention of Lyme borreliosis. For both examples, we provide a background of the particular VBD and the interactions between SDG 3 and other SDGs, which are summarized in Figure 1. A number of dimensions are used to contextualize the assessment of specific synergies and trade-offs, such directionality, place-specific context dependencies, governance, technology, and timeframe (22). To further investigate the nature and dynamics of the interactions, we specify the main targets and key interactions, scored according to the framework and suggest policy options to achieve the intended goal. In the text, we focus only on a subset of the key interactions to illustrate how the scoring framework can be applied in practice. A complete overview of the application's outcome within the framework of the two examples is provided in Tables 1, 2.

**Table 1:** Integrated vector management for dengue control: SDG targets, key interactions among targets, and policy options for prevention.

**Targets(see Figure 1)**	**Key interactions**	Score^*^	**Policy options**
3.3, 3D ← 4.3	Increase knowledge among the population on the relationship between standing water and mosquito breeding and disease transmission. Identify what people already know.	+2^∧^	Invest in effective public campaigns on the control of mosquitoes and the diseases they carry
3.3, 3D ← 6.1	Improving access to safe and affordable drinking water removes the necessity for alternative water storage that serve as potential *Aedes* breeding sites.	+2	Connect water, sanitation and hygiene (WASH) experts with vector experts
3.3, 3D ← 10.1	Progressively achieve and sustain income growth of the bottom 40 per cent of the population at a rate higher than the national average will empower them to become more concerned about mosquito breeding at their premise	+2^∧^	Support vector proof housing programs
3.3, 3D ← 11.6	Improving urban waste management will manage the amount of preventable breeding sites for mosquitoes as well as habitats for other vermin like flies and rats	+2^∧^	Connect the waste management experts with vector experts
3.3, 3D → 12.4	Preventing mosquito breeding sites will diminish the need to use larvicides and the emergency use of adulticides in the environment	+2	Promote sustainable integrated mosquito management
3.3, 3D ← 13.1	Measures to dampen or slow down climate change (mitigation) will also halt the expansion of the *Aedes* distribution	+3	Encourage climate mitigation initiatives
3.3, 3D ← 15.8	Preventing the introduction of invasive *Aedes* species will prevent the expansion of *Aedes*-borne disease areas	+1	Take risk of importing invasive species into account with trade and travel
3.3, 3D → 4.3, 3.3, 3D → 10.1, 3.3, 3D → 11.6	Preventing *Aedes*-borne diseases will positively affect attempts for equity, education and livability of cities	+1^∧^	Invest in integrated health care services

* *Positive interactions are assigned scores +1 (“enabling”), +2 (“reinforcing”), or +3 (“indivisible”), while interactions characterized by trade-offs are scored with −1 (“constraining”), −2 (“counteracting”), and −3 (“canceling”)*.

∧ *The interactions between target 3.3 and 3D with target 4.3, 10.1, and 11.6 are bi-directional, but not symmetrical*.

**Table 2:** Prevention of Lyme borreliosis: SDG targets, key interactions among targets, and policy options for prevention.

**Targets (see Figure 1)**	**Key interactions**	Score^*^	**Policy options**
3.3, 3D ← 4.3.7	Increasing knowledge among the population about the relationship between green spaces, ticks and disease transmission.	+2	When developing green spaces, increase local educational programs on personal protection
3.3, 3D ← 11.7	The development of urban green spaces for leisure and mobility, create new tick habitats and with that risk of contracting Lyme borreliosis increases.	−1	When developing green spaces, make them resilient to ticks
3.4, 3D ← 11.7	With the development of urban green spaces, the well-being of inhabitants will improve	+1	Encourage the development of tick resilient green spaces
3.3, 3D ← 13.1	The development of urban green spaces to counter the heat island effect creates new tick habitats and with that risk of contracting Lyme borreliosis increases.	−1	Encourage the development of tick resilient green spaces
3.3, 3D ← 13.2	Measures to dampen or slow down climate change (mitigation) will also halt the Northern expansion of the tick distribution	+1	Encourage climate mitigation initiatives
3.3, 3D ← 15.2	The implementation of sustainable management of all forests will halt deforestation, restore degraded forests and sustainably increase afforestation globally. However, it will also likely expand the tick habitat.	−2	To minimize the risk of humans to tick bites in forest, decrease the tick densities in nature campsites by keeping the deer out e.g., by means of enclosures

* *Positive interactions are assigned scores +1 (“enabling”), +2 (“reinforcing”), or +3 (“indivisible”), while interactions characterized by trade-offs are scored with −1 (“constraining”), −2 (“counteracting”), and −3 (“canceling”)*.

### Integrated Vector Management for Dengue Control

Dengue (DEN) poses a threat to over 3.9. billion people and its re-emergence has become one of the most serious global health threats (29). However, the more recent spread of Zika virus infections in the world is a more broadly recognized illustration of the re-emergence of mosquito-borne diseases **(SDG 3)**. Driven by ever increasing trade and travel, as well as continuous and progressive urbanization **(SDG11)**, and possibly climate change **(SDG 13)**, the world has been witnessing an increase in diseases that are mainly transmitted by the yellow fever mosquito *Aedes aegypti*, notably DEN, chikungunya (CHIK), and Zika (ZIK). As no effective vaccine or medicine is yet available to prevent or cure DEN, CHIK, and ZIK, disease prevention through vector control is a critical component of disease control, albeit a very challenging one.

*Aedes aegypti*, which thrives primarily in (sub)tropical climate, is a domesticated mosquito species that feeds almost exclusively on humans (30). After the female *Ae. aegypti* has mated and taken a blood-meal, she searches for an aquatic breeding place to lay her eggs. This species lays her eggs preferably in artificial containers, which are often established in urban settings near human habitation (31, 32). *Aedes aegypti* lays her eggs against the vertical side of various kinds of water holding containers and the eggs will hatch upon flooding. The eggs can survive several months of drought (33, 34), and become cryptic for control.

The availability and density of artificial containers are important indicators for the presence and size of *Ae. aegypti* population in a locality (35–37). Satterthwaite showed that the amount of artificial objects can be linked to socio-economic status (38) **(SDG 10)**. The level of access to safe and affordable (drinking) water **(SDG 6)** is inversely proportional to the presence of water storage systems, such as cisterns or rain barrels that are well-known breeding sites of *Ae. aegypti*. Further, in low income areas there is generally more garbage lying around than in high income areas. Random garbage accumulation in a neighborhood can lead to more artificial containers suitable as a breeding ground for *Ae. aegypti* (39, 40) **(SDG 11)**. Neighborhood cleanup constitutes not only an important prerequisite for starting other measures of integrated *Aedes* management, it is to be expected to have a considerable additional positive impact on the health and well-being of the inhabitants. Waste management needs to play a central role in integrated Aedes management that may include chemical control (**SDG12**) in addition to physical and social control measures. While Integrated Vector Management is a public task, it relies, in part, on people to take individual responsibilities **(SDG 4)**. In temperate areas, the risk of establishment of invasive **(SDG 15)** *Aedes* mosquitoes and the pathogens they carry is increasing, due to climate change **(SDG 13)**.

In brief, integrated vector management for dengue control involves, at least, seven SDGs other than the entry goal, SDG3. Within these goals, we identified a total of nine specific main targets (Figure 1). Among these main targets, we recognize eight, all positive, key interactions and suggested an appropriate policy option for each (Table 1). The interactions between target 3.3 and 3D with target 4.3, 10.1, and 11.6 are bi-directional, but not symmetrical. The two SDG3 targets affect the latter targets less, compared to how these targets affect the two SDG3 targets. More specifically, preventing *Aedes*-borne diseases does positively affect attempts of reaching equity, education and livable cities, but we assessed that education (4.3), poverty reduction (10.1) improving urban waste management (11.6) has a more direct and stronger impact on dengue prevention. A complete overview of main targets, key interactions, scores and policy options to reach integrated vector management for dengue control are provided in Table 1.

### Prevention of Lyme Borreliosis

Lyme borreliosis is a VBD caused by an infection with the spirochete *Borrelia burgdorferi s.l*. In Europe, humans primarily become infected through the bite of the sheep tick *Ixodes ricinus*. The transmission cycle of Lyme spirochetes in nature is intricate because, in addition to the tick, it involves several vertebrate host species.

Entering a tick's biotope poses a risk for people since they can acquire a tick bite. To reduce the risk of people acquiring a tick bite, a reduction in either the tick density and/or the exposure is necessary. Understanding, which factors drive tick densities, is an important step in assessing disease risk and formulating intervention strategies (15) (**SDG3**). As *I. ricinus* spends almost its entire life in vegetation, temperature and relative humidity are key requirements for its development, survival and activity and determine their geographic distribution. In conclusion, drivers of tick densities comprise both biotic and abiotic elements of a tick's environment. Land use changes (41) and climate change has had considerable impact on the ticks northern latitude limit (42–45) (**SDG13, SDG 15**). Each of the three active stages (larva, nymph, and adult) of *I. ricinus* seeks a different vertebrate host, attaches, and feeds. The tick detaches when replete and finds a resting place after dropping off the host to digest its blood meal. After this blood meal, ticks molt to the next feeding stage or enter diapause depending on the ambient temperature (46). Adults are the main life stage feeding on (roe) deer, but juvenile ticks (larvae and nymphs) prefer smaller mammals and birds. Although, to some extent, annual fluctuations in rodent densities affect the densities of nymphs the following year, the (local) presence of propagation hosts, mostly (roe) deer, is often the key factor for the presence of moderate tick densities in forested areas (47). Nature and forest corridors not only increase connectivity for vertebrates, but also for ticks and the pathogens they carry (**SDG 15**). When (roe) deer are absent there is a measurable change in abundance of ticks (48), illustrated by significant tick reduction in larger deer exclosures (49). Urban green areas have relatively low tick densities but pose a high risk because of the high human exposure. Over decades urban green areas have been expanded to create more space for nature but also to counter urban warming (**SDG 13**) and to enhance human well-being through access to nature (**SDG 11**) (50).

Effective prevention of Lyme borreliosis from nature requires a sector-transcending approach. It requires an expansion of the integral health policy on education programs (**SDG 4**) with nature policy and nature management (15) (**SDG 15**). This is often inadequate because (the implementation of) these policy areas are located at different administrative levels.

In brief, prevention of Lyme borreliosis involves, at least, four SDGs other than the entry goal, SDG3, comprising a total of eight specific main targets (Figure 1). Among these main targets, we identified six key interactions and suggested an appropriate policy option for each (Table 2). As opposed to the previous example of dengue control, all six key interactions here are unidirectional, in which the three targets of SDG3 are in all cases affected by the identified targets of the other SDGs, but not in reverse. In addition, three of these six interactions are assessed to be negative, including two constraining and one counteracting interaction. A complete overview of main targets, key interactions, scores and policy options to reach prevention of Lyme borreliosis are provided in Table 2.

### Innovation

When following the ICSU framework to move from science to the actual implementation of the SDGs, our perspective on significant issues related to the prevention and control of VBDs and the potential solutions dramatically shifted. The framework helped us identify common goals and targets outside SDG3, the entry goal for the majority of health experts like us. At the same time, the framework illustrates that for each specific problem, even wicked ones, the entry goals and targets interact only with a very limited number of 169 targets underpinning the agenda for sustainable development. We were not as overwhelmed as originally anticipated when starting the implementation of SGDs. Further unlike most integrative initiatives, the framework does not presume that interactions between goals and targets are—for the most part—mutually supporting (22). In contrast, the framework stresses the necessity of identifying and recognizing negative (as well as positive) interactions and facing the challenges. When finding the common intention or goal (SDG) of the various stakeholders involved, joint attention can be devoted to develop policy options that can reach the common goal. For example, by acknowledging that more urban green spaces can potentially increase the tick population and subsequent risk for Lyme borreliosis, more effort can be put in local educational programs on personal preventive measures to protect against increased risk.

An implicit characteristic of any integrated approach is that partnerships are forged (SDG 17), where the focus is placed on sharing, exchanging, collaborating, learning (from each other), reflecting and generating change across disciplines, and sectors in an enabling environment. Such an approach is very helpful to identify the stakeholders for an issue-based approach, in which interactions are mutually beneficial. But greater than this is that when the approach is truly integrative and includes stakeholders across sectors as well as community input, the conversation becomes bidirectional and on some occasions may result in opposing or contradictory views.

Thus, the thesis is confronted by the antithesis, which leads to synthesis in the form of innovation. The SDGs approach not only provides tools to not forget, but also not to be forgotten. It is not a one fit for all approach, but a tool to find old and new partners, to communicate the challenges which force us to be innovative.

## Conclusion

In using the ICSU framework, we arrived at a new way to approach two wicked problems, the control and prevention of dengue and Lyme borreliosis. By identifying key interactions between an entry goal and the targets of all others, we found new approaches for solutions that may enable the creation of novel policy options. The framework provides an easy to reproduce and policy embedded method. It is an innovative suggestion to harness current international policy for improvement of current practice and enable to actually achieve the much proclaimed “paradigm shift” in One Health. In line with ICSU, we hope that this report inspires the development and synthesis of empirical research on interactions across all the SDGs in different parts of the world, and among different scientific and policy communities.

## Author Contributions

MB and GG prepared the manuscript, which was revised primarily by TL. Conceptual and text contributions were made by LT and HS.

### Conflict of Interest Statement

The authors declare that the research was conducted in the absence of any commercial or financial relationships that could be construed as a potential conflict of interest.

## Footnotes

^1^https://ecdc.europa.eu/en/search?s=vector+maps&=

^2^https://www.un.org/sustainabledevelopment/sustainable-development-goals/
